# From genotype to phenotype: Genetic redundancy and the maintenance of an adaptive polymorphism in the context of high gene flow

**DOI:** 10.1002/evl3.277

**Published:** 2022-02-22

**Authors:** Thomas Bataillon, Perrine Gauthier, Palle Villesen, Sylvain Santoni, John D. Thompson, Bodil K. Ehlers

**Affiliations:** ^1^ Bioinformatics Research Center Aarhus University Aarhus 8000 Denmark; ^2^ CEFE, Univ Montpellier, CNRS, EPHE, IRD Univ Paul Valéry Montpellier 3 Montpellier 34293 France; ^3^ UMR AGAP Institut Univ Montpellier, CIRAD, INRAE, Institut Agro Montpellier 34398 France; ^4^ Department of Ecoscience Aarhus University Silkeborg 8600 Denmark

**Keywords:** Ecologically important trait, local adaptation, population genomics

## Abstract

A central question in evolution is how several adaptive phenotypes are maintained within a species. Theory predicts that the genetic determination of a trait, and in particular the amounts of redundancy in the mapping of genotypes to phenotypes, mediates evolutionary outcomes of phenotypic selection. In Mediterranean wild thyme, numerous discrete chemical phenotypes (chemotypes) occur in close geographic proximity. Chemotypes are defined by the predominant monoterpene produced by individual plants in their essential oil. In this study, we analyze the ecological genetics of six chemotypes nested within two well‐established chemical families (hereafter ecotypes). Ecotypes, and chemotypes within ecotypes, are spatially segregated, and their distributions track local differences in the abiotic environment. By combining population genomic, phenotypic, and environmental data from 700 individuals, we show how the genetics of ecotype determination mediates this evolutionary response. Variation in three terpene‐synthase loci explains variation in ecotype identity, with one single locus accounting for as much as 78% of this variation. Phenotypic selection combined with low segregating genotypic redundancy of ecotypes leaves a clear footprint at the genomic level: alleles associated with ecotype identity track environmental variation despite extensive gene flow. Different chemotypes within each ecotype differentially track environmental variation. Their identity is determined by multiple loci and displays a wider range of genotypic redundancy that dilutes phenotypic selection on their characteristic alleles. Our study thus provides a novel illustration of how genetic redundancy of a phenotype modulates the ability of selection to maintain adaptive differentiation. Identifying the precise genetics of the chemical polymorphism in thyme is the next crucial step for our understanding of the origin and maintenance of a polymorphism that is present in many aromatic plants.

**Impact Summary**

Mediterranean aromatic shrubs in the Lamiaceae family are famous for the diversity of their scents, and are used both as culinary herbs and in the pharmaceutical industry. Molecules—primarily monoterpenes—contained in their essential oil confer the different scents. These monoterpenes can provide a range of ecological functions including protection against herbivores and temperature and drought stress via their antioxidant properties and their ability to maintain functional cell membranes under stress. These protective properties vary among monoterpenes and the identity of the predominant monoterpene produced by a plant defines distinct chemical types (chemotypes). In its natural distribution, *Thymus vulgaris* (wild thyme) harbors several distinct chemotypes whose spatial variation is associated with a variation in highly localized climatic conditions. The predominant monoterpene is either a phenolic or a nonphenolic type. These two types are ecotypes with adaptation to sites that can experience either mild or, in contrast, early, extreme winter freezing. Chemical variation also exists within phenolic (two chemotypes) and nonphenolic (four chemotypes) ecotypes and six chemotypes frequently coexist on a landscape scale. We studied the ecological genomics of these chemotypes within an area of 25 km^2^ in the South of France. To understand how selection shapes the distribution of this polymorphism, it is essential to know the genetic basis underlying chemical variation. Here, we show that genetic variants located in genes of the monoterpene pathway explain a large fraction of chemotype variation, and that the genetic determination of ecotypes can explain why close populations contain distinct ecotypes despite extensive gene flow. This can also explain how ecotypes quickly responded to recent climate change. Identifying candidate genes underlying chemotype variation in thyme will provide the opportunity to dissect how this crucial chemical polymorphism has arisen and diversified in aromatic plants.

How to maintain discrete stable phenotypes within a single species is a long‐standing conundrum for ecological and evolutionary theory. Theories for the maintenance of different phenotypes abound and besides trivially re‐introducing new types by mutation several mechanisms have been proposed (Debarre and Lenormand 2011). Negative frequency‐dependent selection, a fitness advantage to a locally rare type, automatically solves the maintenance problem. Self‐incompatibility alleles in angiosperms are a known example, where numerous (mating) types are encoded by a single (super) locus; but examples of many (>2) adaptive discrete phenotypes maintained by this mechanism are few and far between; the Rock‐Scissor‐Paper dynamics in lizard color morphs is one of the rare examples (Sinervo and Lively 1996). Another solution involves environmental heterogeneity, with selection maintaining locally adapted alleles conferring alternative phenotypes (Colosimo et al. 2004; Barrett et al. 2019). Many models also illustrate how local selection can maintain phenotypic differentiation despite drift and the swamping effect of migration (Savolainen et al. 2013; Yeaman 2015).

The genetic basis of phenotypic variation is critical here. Whether one or many loci encode phenotype variation and whether these loci segregate independently or as a single entity (supergene) determines the likelihood of local selection maintaining phenotypic variation in the face of gene flow. Theory suggests that the number of genotypes that, at a given time, segregate in a population and are phenotypically redundant, so called segregating redundancy, is a critical parameter governing both the predictability of evolution (Chevin et al. 2010; Lässig et al. 2017) and the capacity of locally heterogenous selection to maintain different adaptive phenotypes (Láruson et al. 2020). The level of segregating (genotypic) redundancy determines in fact the intensity of selection on individual alleles. Higher redundancy (several different genotypes can produce the same phenotype) makes it more difficult to either fix or eliminate individual alleles compared to situations where alternative phenotypes are determined by one or a few genotypes. Redundancy may also help maintain phenotypic differentiation in the face of high gene flow, as several genotypes producing the same adapted phenotype can contribute to withstand swamping due to the immigration of divergent alleles (Láruson et al. 2020).

Studies on the genetics of ecologically important phenotypes have, often conveniently, focused on cases of a single locus with major phenotypic effect: for example, *Eda* locus in Sticklebacks (Colosimo et al. 2004) and *Agouti* in Mice (Barret et al. 2019). As Stinchcombe and Hoekstra (2008) and Sella and Barton (2019) point out, this is at odds with the view that phenotypic variation in fitness‐related traits is controlled by genetic variation at a few (2‐5) loci with major effects (oligogenic traits) or many (>10) loci with small individual effects (polygenic traits). Several decades of quantitative genetics on the model plant *Arabidopsis thaliana* illustrate the challenges of studying the genetic architecture of polygenic traits of ecological importance such as flowering time (Zan and Carlborg 2019) or rosette growth (Wieters et al. 2021). In such cases, many crosses (F2 or recombinant inbreed lines) or numerous accessions in genome‐wide association studies are needed to detect genetic variants with small effects.

Studying traits spanning a continuum of genetic architectures is still, however, much needed to more broadly understand how phenotypic variation is maintained and how natural selection can change ecologically important traits (Láruson et al. 2020) and thereby mediate evolutionary response. Here, we focus on a key ecological trait with oligogenic inheritance: the chemical composition of volatile oils in aromatic plants. This type of variation allows us to directly relate genotypes to alternative phenotypes while moving away from a single locus case. In doing so, we have been able to study the level of genetic redundancy of phenotype conditions and how phenotypic selection translates into selection on individual alleles.

The evolutionary ecology of the Mediterranean aromatic wild thyme (*Thymus vulgaris*) has been studied extensively for almost 50 years. *Thymus vulgaris* contains at least seven distinct chemical phenotypes (called chemotypes) (Saez and Stahl‐Biskup 2002), each chemotype producing a different predominant monoterpene, which represents 50–80% of the chemicals in the essential oil (Thompson et al. 2003). In wild thyme, the predominant monoterpene is either a phenolic or a nonphenolic molecule. This distinction defines two ecotypes that track variation in the abiotic environment: the phenolic ecotype occurs in areas with milder winters and more severe summer drought than sites where the nonphenolic occurs (areas with occasional severe early winter freezing [Gouyon et al. 1986; Thompson et al. 2007]). A local population is typically composed of one ecotype with one or two chemotypes; when two chemotypes coexist, they are most often of the same ecotype.

Long‐term reciprocal transplant experiments have confirmed the adaptive nature of phenolic and nonphenolic ecotypes to the abiotic environment. At nonphenolic sites, fitness differences were such that survival of nonphenolic transplants was more than twice as high as that of phenolic transplants during a winter that involved an early severe freezing event. At phenolic sites, the survival of phenolic transplants was likewise twofold higher than the survival of nonphenolic chemotypes following the extremely hot summer of 2003 (Thompson et al. 2007). Interestingly, in the study area we present in this article, the lack of severe winter frost in the last three decades is associated with an increase in the frequency of the phenolic ecotype and serves as a dramatic example of rapid phenotypic response to ongoing climate change (Thompson et al. 2013).

In this study, we examine how genetic determination of chemotypes and environmental variation contributes to maintain this striking polymorphism. Previous crossing experiments demonstrated that chemotype variation is strongly heritable and likely controlled by the segregation of genetic variants at relatively few (5‐6) independent loci with strong epistatic interactions (Vernet et al. 1986). More recent studies point to an effect of gene dosage with quantitative effects within chemotypes (Thompson et al. 2003). However, this polymorphism has never been studied using a genomic approach, and the genetic polymorphism underlying chemical variation remains to be identified. Here, we combine population genomic data with phenotypic and environmental data from 21 populations located at geographically close sites. Together, these naturally occurring populations harbor six distinct chemotypes in two main ecotypes (Figure 1A). Having so much phenotypic variation present in geographically close sites is a unique situation: It allows to study single nucleotide polymorphism (SNP) phenotype association and environmental selection on many different types without the confounding effect of population genetic structure that often arises at larger spatial scales.

**Figure 1:**
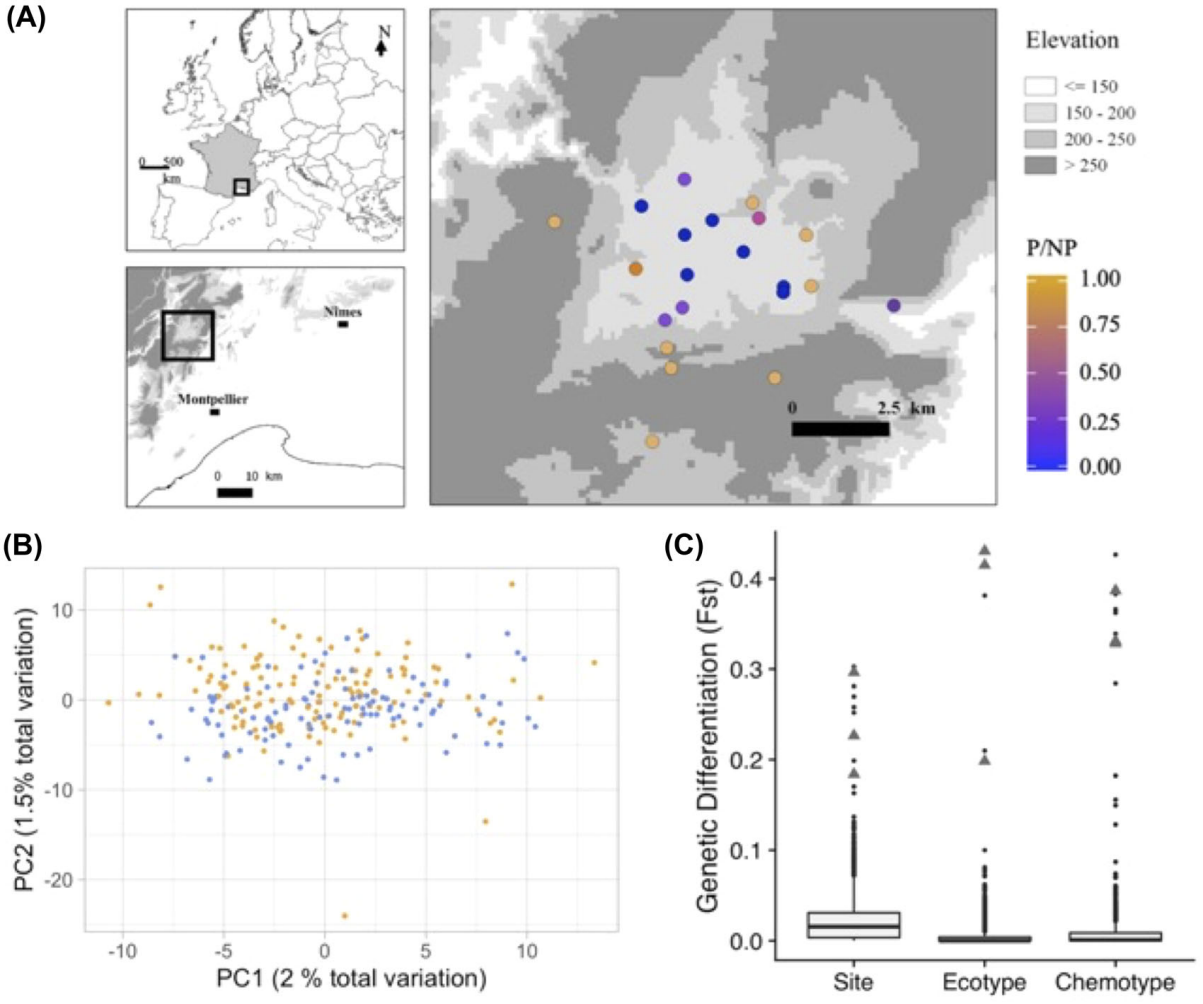
Overview of locations and genetic differentiation. (A) Location of the 21 populations used for the study. Every population (dot) is colored to represent the composition (P/NP) of proportion of phenolic ecotypes in each population, ranging from purely phenolic (P: orange) to purely nonphenolic (NP: blue). Shades of gray depict elevation (altitude in meters above sea level). (B) First (PC1) and second (PC2) principal components of SNPs variation. Every individual (dot) is colored by its ecotype identity (blue dots: nonphenolic; orange dots: phenolic). (C) Boxplots depicting the distribution of *F*st (*n* = 3920 SNPs) among sites (21 populations), ecotypes (phenolic vs. nonphenolic), and among chemotypes (six different individual chemotypes). Triangles ticks denote the *F*st values for three SNPs used to build the genotype to ecotype map (Figure 3A). Note that other SNPs with high *F*st display no phenotype associations.

## Materials and Methods

### PLANT MATERIAL

In June 2016, 21 sites were sampled within an area around the village of Saint‐Martin‐de‐Londres (Figure 1A). The sampling of sites was based on prior knowledge of spatial chemotype variation in this area. Sites were chosen to maximize the diversity of abiotic environment as well as to span the known spatial gradient of chemotype diversity in the region. At each location, 12 individuals were randomly sampled, while ensuring that individuals were growing at least 1.5 m apart. For each individual, fresh leaves were gathered in sealed plastic bags and stored in cooling bags until returned to the labs where samples were kept at −80°C until DNA extraction. In addition, leaves (shoot tip 2 cm) for GC‐MS analysis were sampled in 1 mL of methanol. After 24 h, the methanol extractions were transferred to fresh tubes and stored at −80°C until analyzed on GC‐MS.

### PHENOTYPING: CHEMOTYPES

The chemotype of the plants sampled from the 21 sites (252 individuals) was determined as the identity of their predominant monoterpene (either geraniol, alpha‐terpineol, thuyanol, linalool, carvacrol, or thymol) determined from GC‐MS. In brief, methanol extracted samples were analyzed on a Shimadzu GCMS‐QP21010 fitted with a flame ionization detector and a fused silica capillary column SLB‐MS (30 × 0.25 mm; 0.25‐μm thickness). Helium was used as a carrier gas. The compounds were identified by comparing retention time and mass spectra with standards from Mass Spectral Library.

To increase information on chemotype frequency within and among sites, we added available data on individual plants sampled from the same sites that were chemotyped just few years prior to our sampling. Our dataset representing chemotype frequency at the 21 sites thus consists of chemotype identity of 702 individual thyme plants: 252 individuals sampled in 2016 in this study, and 450 individuals from 2013 (see Thompson et al. 2013 for details).

### ENVIRONMENTAL VARIABLES

#### Soil variables

At each site, 1 kg of soil was collected with two to three different representative samplings within each site. Soils were dried at 40°C for 48 h, sieved at 2 mm, and stored in a cool room prior to analysis. Water retention potential was calculated as the percentage of water lost after drying a wet soil for 48 h at 40°C. Water retention capacity was calculated as the percentage of water remaining in this previously 40°C‐dried soil by a repeated drying of the sample at 110°C for 5 h. Organic matter was estimated as the percentage of matter lost after burning a dried sample at 500°C during 5 h.

#### Temperature variables

At each site, two data loggers were placed from January to February and from May to September. Data loggers were secured on branches of thyme or other small shrubs about 20‐40 cm above the ground. Data loggers recorded temperature every 30 min. A few data loggers were lost in the January‐February period (Figure S1). After collecting data loggers, the recorded temperature was extracted and, for each site, used to create the temperature variables.

The individual variables used were percentage water in soil, soil water retention and percentage organic matter in the soil (“pctsoilwater,” “pctwaterret,” “pctorgmat”), the mean daily minimum and maximum temperature in winter (“mint”, “maxt”), minimum temperature in the coldest month (“mintjan”), the number of days where moderate to strong freezing was recorded (below −8°C, “freezemoderate”), the mean daily minimum and maximum temperature in the summer (“maxsumt,” “meansumt”), the number of days exceeding 40 or 45°C (“dayabove45,” “dayabove40”), and the mean summer daily temperature amplitude (“meansumamplit”). For summer temperature, we omitted the data recorded between 12:00 p.m. and 04:00 p.m., as direct sunlight may cause data loggers to record unusually high temperatures not representative of the site. We checked that omitting these time intervals did not alter the ranking of the warmest to coldest sites.

### LOCUS‐TARGETED GENOTYPING

We used a locus‐targeted genotyping by sequencing procedure. A set of 20,000 DNA baits (myBaits, Arbor Biosciences) was designed to target candidate genes previously identified by transcriptome sequencing (Mollion et al. 2018), known genes encoding enzymes of the monoterpene biosynthesis pathway previously identified in *T. vulgaris* or close relatives (GenBank Accessions: JF940523.1, KM272332.1, KM272331.1, KU699534.1, KU699532.1, KU699531.1, KU699530.1, KU699529.1, KR920616.1, KC461937.1, JX946358.1, JX946357.1), along with 300 anonymous targets that were genes identified by transcriptome sequencing but not expected to be associated to phenotypes.

Plant DNA was extracted from 15 mg of fresh young leaves. Genomic library preparation for multiplexed individuals and enrichment step by bait capture follow published protocols (Mascher et al. 2013) with some modifications. Briefly, for each genotype a genomic barcoded library was built; 48 barcoded libraries were mixed together for the capture by hybridization step. To increase the specificity of the enrichment step, we used a capture, washing, and recapture procedure. Captured DNA was sequenced by sets of 144 genotypes on two runs of HiSeq 3000 Illumina NGS sequencer (full protocol available as *Methods* in the Supporting Information).

### READ ALIGNMENT AND SNP CALLING

All reads were mapped using BWA (Li and Durbin 2009) against a reference that comprises our full de novo transcriptome assembly (Mollion et al. 2018) and GenBank reference sequences from candidate genes (see list of accessions above). Each final sequence in the reference was tagged as either “full candidate” (FC: transcriptome candidates of the GenBank sequences), “anonymous target” (AT), or “background” (BG). All bamfiles were sorted and indexed, and PCR and optical duplicates were marked using samtools before SNPs were called using read2snps version 2.0 (Gayral et al. 2013) and GATK version 3.8 (Van der Auwera 2013) using default settings. A set of individuals that were independently baited/sequenced and processed were used to assess the overall reliability of the SNP calling (see *Methods* in the Supporting Information and Table S1).

### PRINCIPAL COMPONENTS OF SNPs VARIATION AND GENETIC DIFFERENTIATION

To summarize the patterns of genetic variation, we performed a principal component analysis (PCA) on the genotypes scores of all common SNPs. Each individual SNP genotype score was centered and reduced before performing a PCA. A probabilistic PCA, as implemented in the R package *pcaMethods* (Stacklies et al. 2007), was used and 10 PCs were computed. The PCs were used for accounting for population structure when detecting SNP‐phenotype (ecotype or chemotype) and SNP‐environment associations.

To quantify genetic differentiation between population, ecotype, and chemotypes, we estimated *Fis* and *F*st via Weir and Cockerham estimators using the R package *NAM* (Xavier et al. 2015) on the set of nonredundant SNPs. A set of nonredundant 3920 SNPs was obtained using the quality control function snpQC in the *NAM* package (requesting less than 50% missing data per SNP, and excluding highly correlated SNPs, threshold 0.98). If two SNPs had at least 98% identical genotypes across all individuals called, they were considered as redundant.

### SNPs‐ECOTYPE AND SNPs‐CHEMOTYPE ASSOCIATIONS

We tested for associations between the nonredundant 3920 SNPs and chemotype (or ecotype) identity across the sample of individuals of the 21 sites. We evaluate association by comparing the fit of two logistic regression models. The first is a background model (M_0_) where five PCs of SNPs variation are used to predict chemotype:

M_0_: binary chemotype ∼ PC1 + PC2 + PC3 + PC4 + PC5.

We expect M0 to have some predictive power because of small genetic differentiation between populations and the fact that populations differ for their ecotype (chemotype) composition.

The second model is a SNP model (M_1_) that also uses a single SNP genotype (coded as 0, 1, 2) as predictor:

M_1_: binary chemotype ∼ PC1 + PC2 + PC3 + PC4 + PC5 + SNP.

Models were fitted by maximum likelihood for each SNP assuming a binomial distribution for the binary phenotype identity (*Methods* in the Supporting Information).

### SEGREGATING REDUNDANCY UNDERLYING DISCRETE PHENOTYPES

A set of *T* (possibly multilocus) genotypes *G_i_*, *i* = 1, …, *T*, segregate with genotypic frequency $p_{i}$ ( $\sum_{i\;=\;1}^{T}p_{i}=1)$. A set of *K* discrete phenotypes $P_{j}$, j = 1, …, K, are “encoded” by the set of T genotypes *G*_1_, …, *G_T_*. We define a coefficient $w_{ij}$ that, for each pair $\left(G_{i},P_{j}\right)$, measures how strictly phenotype $P_{j}$ is determined (encoded) by genotype $G_{i}$. The genotype to phenotype map (encoding) can be genetically strict or loose depending on the genetic architecture of the K phenotypes. We use the conditional probability that genotype $G_{i}$ will produce phenotype $P_{j}$ to measure how strict is the genotype to phenotype map for a given *P_j_*:

$$w_{ij}\;=Prob\;\left(P_{j}|G_{i}\right)$$

Note that $w_{ij}\;\;=\;\;1$ captures the case where individuals with a specific genotype *G_i_* always produce phenotype *P_j_*.

We measure segregating redundancy using a statistic analogous to the effective number of genotypes (akin to Nei's effective number of alleles at a locus defined via Nei's heterozygosity). We use the phenotype‐specific weighting $w_{ij}$ when calculating the effective number of genotypes.

We calculate a (weighted) genotypic diversity that is segregating for determining each phenotype $P_{j}$ as

$$\;D_{j}=\;1-\sum_{i\;=\;1}^{T}\psi_{ij}^{2},$$

where $\psi_{ij}=\frac{p_{i}w_{ij}}{\sum_{i}p_{i}w_{ij}}\;$.

The $\psi_{ij}$s are estimated as the frequency of each genotype $G_{i}$ weighted by probability $w_{ij}$ that it will produce an individual with phenotype $P_{j}$. If all possible genotypes have same probability to produce $P_{j}$, then $\psi_{ij}=p_{i}$.

The segregating redundancy for phenotype $P_{j}$, $SR_{j}$, is the effective number of genotypes encoding $P_{j}$:

$$SR_{j}=\frac{1}{1-\;D_{j}}\;.$$

If *T* genotypes can encode phenotype $P_{j}$, $SR_{j}$ will be maximal and equal to T when all T genotypes are equally frequent and encode $P_{j}$ with identical probability ($\psi_{ij}=1/T$ for all i).

If few genotypes $G_{i}$ encode $P_{j}$ with high probability or genotypes have unbalanced frequencies, then $SR_{j}$ will be lower. The minimum $SR_{j}$ is 1: this is the limit situation where despite the fact that T genotypes coexist in the population only one genotype is effectively “encoding” phenotype $P_{j}$. For a given amount of phenotypic selection affecting phenotype $P_{j}$, $SR_{j}$ measures how much phenotypic selection “percolates” at the genotype(s) level. The SNPs‐ecotype and the SNP‐chemotype associations detected were used to define multilocus genotypes. We use observed frequency of genotypes and discrete phenotypes to estimate $p_{i}$s, the $w_{ij}$, $\psi_{ij}$, and $SR_{j}$s. Resampling (500 bootstraps at the individual level) was used to obtain SEs around $SR_{j}$ estimates.

### ECOTYPE/CHEMOTYPE‐ENVIRONMENT ASSOCIATION

To characterize environment variation, we performed a PCA on the centered and reduced environmental variables measured at the 21 sites. All environmental variables were centered and normalized (to unit variance) before PCA. Principal component (PC) 1, PC2, and PC3 account for 45%, 28%, and 12%, respectively, of the total variation measured across sites. PC1 correlated with variables measuring winter freezing and PC2 strongly correlated with the number of days exceeding 40°C (Figure S1).

We used a (multinomial) logistic regression where counts of individuals in each ecotype (phenolic C, T vs. nonphenolic G, aT, U, L) or all six chemotypes (G, aT, U, L, C, T) at each site (using all 702 chemotyped individuals) are used as response variables and the PCs of environment variation used as predictors (De Mita et al. 2013). Likelihood ratio tests comparing the deviance of models were used to obtain statistical significance for the effect of the different (principal) components of environment variation on ecotype/chemotype distribution. Likelihood ratio tests were conducted assuming that differences in likelihoods are approximated by a chi‐squared distribution (with *P* degrees of freedom if models differ for *P* fitted parameters). To quantify the amount of variation accounted for by each model, we used the pseudo *R* ^2^ computed as 1 – Dev(M*_i_*)/Dev(M_0_), where Dev(M*_i_*) is the deviance of model M*_i_* and Dev(M_0_) is the deviance of a model with no predictors (merely fitting an intercept).

### SNPs‐ENVIRONMENT ASSOCIATION

We used a similar logistic regression framework as the one described above for SNP‐phenotype associations. Instead of using phenotypes as response variables, we use variation of SNP frequency from site to site as a response variable and test whether SNPs covary with the environment measured using Environmental PC1 and PC2 (*Methods* in the Supporting Information).

## Results and Discussion

### LOW GENETIC DIFFERENTIATION AMONG POPULATION DESPITE STRIKING PHENOTYPIC DIFFERENCES

Our genomic data were produced by the resequencing of target genomics regions (after enrichment of genomic DNA) in 252 individuals. The target regions are candidate genes of the monoterpene biosynthesis pathway (14 genes) and a larger set of anonymous genes (384 genes) that were previously characterized by transcriptome sequencing (Mollion et al. 2018). Targeted resequencing yielded an average coverage of 2.46 per individual in targeted regions and 0.007 in the rest of the mappable reference genome. This corresponds to a 300‐fold coverage increase relative to the coverage we would have achieved by whole genome resequencing without bait capture of targeted regions.

We focused on 3920 SNPs that all had a frequency of the rarest allele >0.1. This includes 98 SNPs anchored in genes of the monoterpene biosynthesis pathway. Using a set of independent technical replicates (identical DNA but independent library preparation, sequencing, reads mapping, and SNP calling), these SNPs were shown to be highly reproducible genetic variants (*Methods* in the Supporting Information and Table S1). All the analysis presented here rely on this set of SNPs.

Genetic differentiation among sites, measured by average genome differentiation at the 3920 SNPs, is very low (*F*st between sites: 0.021 ± 0.003), and is even lower between ecotypes (*F*st between ecotypes: 0.004 ± 0.0002; Figure 1B,C). This confirms, at a genome‐wide scale, previous interpretations based on analyses of allozyme loci (Thompson 2020). A striking spatial structure in the distribution of ecotypes is maintained over a small geographic scale despite very low genetic differentiation among populations of the two ecotypes. Within sites, deviation from random mating, as measured by *F*is, is negligeable (mean *F*is = 0.01 ± 0.005).

**Figure 2:**
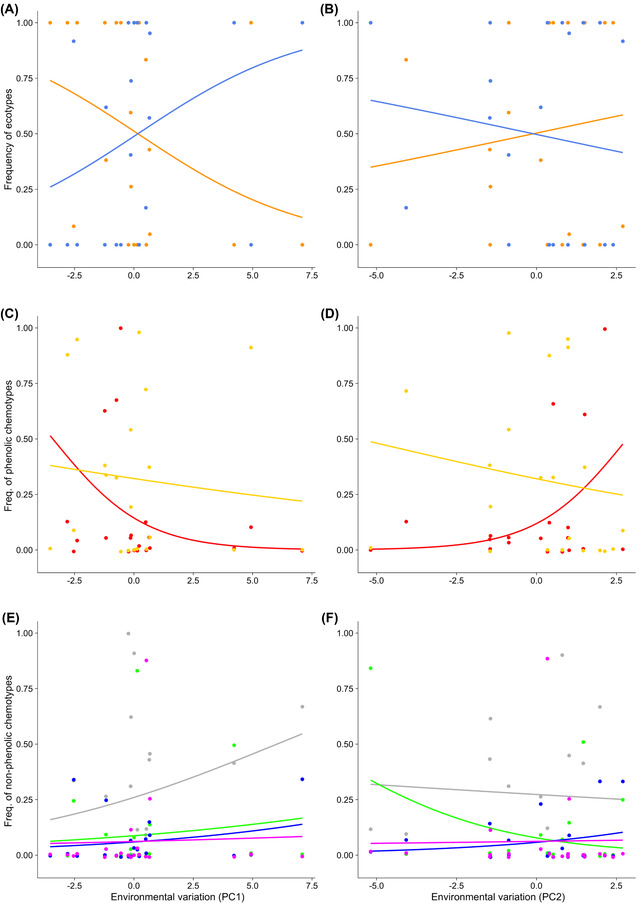
Phenotype‐environment reaction norms. Each dot represents the observed population frequency of phenotypes (either ecotypes in panels A and B or chemotypes in panels C‐F) as a function of the environment measured in a given site (measured through either PC1 or PC2 of a PCA on environment variables). Lines indicate predictions from logistic regression. Color of line indicates ecotypes’ identity (in panels A and B): phenolic (orange), nonphenolic (cornflower blue), and chemotypes (in panels C‐F): G (magenta), aT (blue), U (green), L (gray), C (red), and T (yellow). Note that in panels A and B, observed frequencies (and fits) of phenolic and nonphenolic are mirror image as these are mutually exclusive phenotypes.

We quantified the relative contribution of local genetic drift and environmental variation to variation in phenotypes across populations using PCA of the SNP variation as predictors of differentiation between populations (local genetic drift) and PCs of abiotic variation as predictors of environmental variation. A first model (Genetic Drift) is fitted using only SNPs PCs as predictor of phenotypes composition. A model featuring both SNPs and environment PCs (Genetic Drift + Environment) is then fitted and the extra fit provided by this model is our measure of the importance of the environment (beyond any effects of local drift). Examining how these two sets of predictors explain variation across populations on ecotype distribution showed a large effect of environmental variation (Table 1). Repeating the same analysis for individual chemotypes revealed that a sizeable proportion (10–20%) of the distribution of individual chemotypes across populations is explained by environmental variation, with a comparatively smaller effect of genetic drift (Table 1). Individual chemotypes within an ecotype are not interchangeable and differentially track environmental variation (Figure 2; Table 1).

**Table 1:** Association of ecotypes and chemotypes with environment after accounting for genetic drift

Models Fitted^a^	All Chemotypes	Ecotypes	G	aT	U	L	C	T
Dev(Null)	853.30	103.66	62.36	52.64	69.01	85.00	102.64	104.36
Dev(Genetic Drift)	737.16	95.71	35.78	46.16	54.24	65.95	76.39	98.07
Dev(Genetic Drift + Environment)	569.84	79.91	34.19	28.07	28.72	56.19	51.30	83.31
*R* ^2^ genetic drift	0.14	0.08	0.43	0.12	0.21	0.22	0.26	0.06
*R* ^2^ genetic drift + environment	0.33	0.23	0.45	0.47	0.58	0.34	0.50	0.20

a Models fitted: “Genetic Drift” model uses five PCs of SNPs variation and the “Genetic Drift + Environment” model uses five PCs of SNPs and three PCs of environmental variation to predict the distribution of all chemotypes jointly (multinomial logistic regression). Individual ecotype or chemotype: G, aT, U, L, C, and T. Dev() refers to the deviance of each model.

*R* ^2^ are pseudo *R* ^2^ quantifying the percent of variation explained by genetic drift and by genetic drift and environment combined. These are calculated as 1 – Dev(Genetic Drift)/Dev(Null), and as 1 – Dev(Genetic Drift + Environment)/Dev(Null), where Dev() is the deviance of each model.

### ECOTYPE VARIATION ASSOCIATES TIGHTLY WITH GENETIC VARIATION AT THREE CANDIDATE LOCI

Using a logistic regression framework accounting for the underlying weak population structure (Figure 1B,C), and a conservative Bonferroni correction to account for multiple testing (requesting *P* < 0.05/3920 at individual SNPs), we show that ecotype identity (phenolic vs. nonphenolic) strongly associates with variation at three genetic loci.

**Figure 3:**
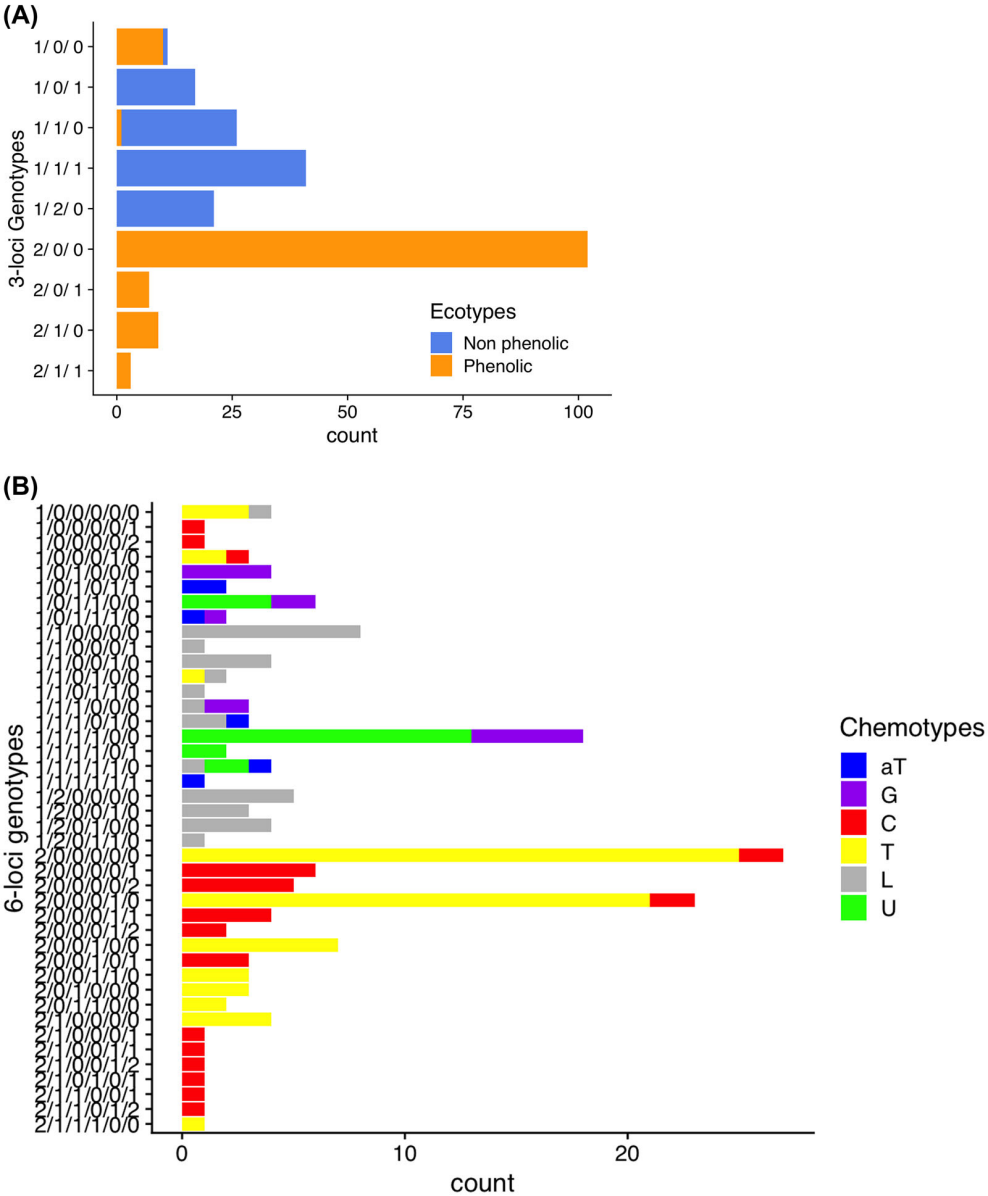
Genotype to phenotype maps: Ecotypes and chemotypes. (A) Genotype to ecotype map (based on *n* = 237 individuals). The three‐loci genotypes are built using sequentially the three SNPs with the strongest associations (Table 2, Panel A). Count refers to the number of individuals in each category. 0, 1, and 2 denote the three possible genotypes at each SNP (0 and 2 are genotypes homozygous for the reference and alternative allele; 1 is the heterozygous genotype). (B) Genotype to chemotype map (based on *n* = 179 individuals). Six‐loci genotypes are built using the six SNPs with the strongest associations (using sequentially the SNPs reported in Table 2, Panel B). Counts refers to the number of individuals in each six‐loci genotype category. Colors indicate chemotype identity.

An SNP in one single gene—homologous to a linalool synthase—explains 78% of the observed ecotypic variation between individuals. SNPs located within two additional genes (homologous to two gamma‐terpinene synthases) are also strongly associated with ecotypes (both *P* < 10^−8^), but with more modest apparent effects (Figure 3A; Table 2). The three SNPs displaying the strongest associations (Table 2A) exhibit some statistical pairwise association (*r* ^2^ between genotypes ranging from 0.1 to 0.42). All pairwise genotype disequilibria involving these three SNPs are statistically significant (by a Fisher's exact test, all *P*‐values <0.00002), suggesting physical linkage between the three genes; although we do not have a whole genome assembly to estimate the actual physical distance between these genes. The presence of mild to strong statistical associations between the top three SNPs precludes us from parsing the associations we detect into reliable SNPs‐specific phenotypic effects and estimating how many causal variants (at least one and up to three) may be responsible for these SNP‐ecotypes associations. Alternatively, the maintenance of strong genotypes disequilibrium we observe could also reflect epistatic selection acting to maintain associations between these three SNPs genotypes.

**Table 2:** Summary of top SNP‐ecotype and SNP‐chemotype associations

Panel A: Top three SNPs‐ecotype associations			
Contig/SNP position on contig	Gene function (by homology)^a^	*R* ^2^ ^b^	*P‐value*
Contig71000/648	Linalool synthase	0.78	6.42 × 10^–54^
Contig12377/126	Gamma‐terpinene synthase	0.45	9.89 × 10^–31^
KR920616.1/174	Gamma‐terpinene synthase	0.18	4.02 × 10^–13^

Panel B: Top six SNPs‐chemotype associations. Numbers in each chemotype column refer to the *P*‐value for a test of SNP‐chemotype identity associations.							
		Individual chemotype					
Gene/SNP position on gene	Putative gene function	C	T	G	aT	U	L
Contig71000/648	Linalool synthase	2.68 × 10^–9^	3.06 × 10^–24^	8.59 × 10^–5^	4.95 × 10^–6^	5.13 × 10^–8^	7.9 × 10^–23^
Contig12377/126	Gamma‐terpinene synthase	0.000264	1.59 × 10^–19^	0.205	0.246	0.125	1.52 × 10^–30^
KR920616.1/174	Gamma‐terpinene synthase	0.0017	8.04 × 10^–8^	5.37 × 10^–10^	2.62 × 10^–10^	6.44 × 10^–14^	1.6 × 10^–5^
JX946358.1/1452	Sabinene hydrate synthase	0.0195	0.00845	0.181	0.25	1.8 × 10^–12^	0.151
KC461937.1/1279	Alpha terpineol synthase	0.454	0.542	0.045	1.22 × 10^–7^	0.00998	0.869
KM272332.1/359	Limonene‐3‐hydroxylase	2.36 × 10^–27^	5.01 × 10^–13^	0.0382	0.968	0.269	0.00065

^a^ Most probable gene function inferred via sequence homology at the nucleotide level for contigs: Contig71000 has strong homology (96.4% and 97.1% nucleotide identity) to *Thymus vulgaris* terpene synthase sequences (tps3 96.39% GenBank accession JX997982.1, tps4 97.08% accession JX997983.1); Contig12377 has strong homology to a *Thymus vulgaris* γ‐terpene synthase 2 (tps2) mRNA, complete cds (2699 nucleotides with 99.53% sequence identity to JX997981.1, 99.46% to MH686200.1).

^b^ *R* ^2^: individual (pseudo) *R* ^2^ associated with the effect of a variant (obtained from the deviance of a model including five PCs of SNPs variation + a candidate SNP relative to a null model only including five PCs of SNPs variation).

A model combining these three loci genotypes accounts for virtually all ecotype variations as illustrated by the near perfect co‐segregation between the three loci genotypes and ecotype identity (Figure 3A). This is consistent with the heritable and oligogenic control of the polymorphism (Vernet et al. 1986). For two of these loci, no individuals are homozygous genotypes for the rarest allele detected, but these loci do not exhibit abnormal *F*is values (Table S2). Independent sequencing and genotyping of 23 individuals confirms that this is not an artifact of poor genotyping at these SNPs (*Methods* in the Supporting Information and Table S1). The three SNPs displaying the strongest statistical association with ecotype variation are also among the SNPs exhibiting the highest genetic differentiation among sites (Figure 1A), consistent with the interpretation of local environmental selection on ecotypes.

### CHEMOTYPE VARIATION IS MORE DIFFUSE AND HARBORS CONSIDERABLE GENOTYPIC REDUNDANCY

The chemotypes show very low overall genetic differentiation (mean *F*st among chemotypes = 0.007 ± 0.0003; Figure 1C). We tested for associations between genetic variants (SNPs) and individual chemotype identity. When using the logistic regression described above along with the same conservative threshold for significance (*P* < 0.05/3920), we detected the same three variants already associated with ecotype differences, as well as additional SNPs located in three independent genes that strongly associate with individual chemotypes. The SNPs that display the strongest associations with individual chemotype identity are located within six genes all identified as homologs for previously reported enzymes of the monoterpene biosynthetic pathway (Table 2). Collectively, 45% of the variation in chemotype identity can be explained by SNP variation in these six loci (Figure 3B). The six SNPs that have the strongest association with chemotypes were also among the SNPs that showed most differentiation among sites (Figure 1C), suggesting that although gene flow among populations is high, heterogeneous selection imposed by the local abiotic environment contributes to spatial segregation of chemotypes within ecotypes.

### MEASURING SEGREGATING GENETIC REDUNDANCY

We propose a measure of segregating redundancy inspired by recent work on genetic redundancy (Láruson et al. 2020), which primarily focused on continuous and potentially highly polygenic phenotypes. Here, we quantify segregating redundancy for discrete phenotypes. The discrete phenotypes can be either alternative ecotypes (phenolic and nonphenolic) or individual chemotypes (G, aT, U, L, C, and T). Given that selection operates on phenotypes, a segregating genetic redundancy measure should capture how many segregating entities (alleles at a single locus or in our case multilocus genotypes) underly phenotypic variation.

We measure segregating redundancy as the effective number of genotypes underlying each discrete phenotype, akin to an effective number of alleles at a locus. The effective number of genotypes is estimated using frequencies of alternative genotypes that can produce a given phenotype weighted by how much a genotype is determining the phenotype (methods). We use the observed phenotype‐specific weighting of each genotype when calculating the effective number of genotypes (Table 3).

**Table 3:** Segregating redundancy (SR) of ecotypes and chemotypes

**Ecotypes**	SR	SE(SR)
Phenolic	1.64	0.13
Nonphenolic	3.53	0.25
**Chemotypes**		
G	3.31	0.66
aT	2.89	0.86
U	2.26	0.51
L	6.57	1.12
C	8.18	1.38
T	4.36	0.56

*Note*: Segregating redundancies (SRs) of ecotypes and chemotypes are computed using the frequencies of the three and six‐loci genotypes (built using the SNPs reported in Table 2, Panels A and B) and the genotypes to phenotypes relationships pictured in Figure 3A,B. SEs are based on 500 bootstraps at the individual level.

Very few distinct genotypes account for ecotype variation (Figure 3A). Accordingly, segregating genetic redundancy is low with 3.53 and 1.64 genotypes effectively determining the nonphenolic and phenolic ecotype, respectively (Table 3). Low segregating redundancy means in turn that strong phenotypic selection translates into strong selection on individual genotypes. This explains how differentiation of ecotypes among populations in different abiotic environments can be maintained despite considerable gene flow across the spatial scale we study.

In contrast, the number of effectively segregating genotypes of each chemotype is considerably higher than for ecotypes (Figure 3B; Table 3): the “U” chemotype for instance has the lowest estimated redundancy (SR = 2.25 ± 0.51), whereas the “C” chemotype has a segregating redundancy that is more than three times higher (SR = 8.18 ± 1.38). We emphasize that, for the individual chemotype identity, genetic redundancy is underestimated as the six‐loci genotypes we use (Figure 3B) can only account for 45% of observed individual variation in chemotype identity.

### EFFECTS OF SELECTION DEPEND ON PHENOTYPE GENETIC ARCHITECTURE AND REDUNDANCY

**Figure 4:**
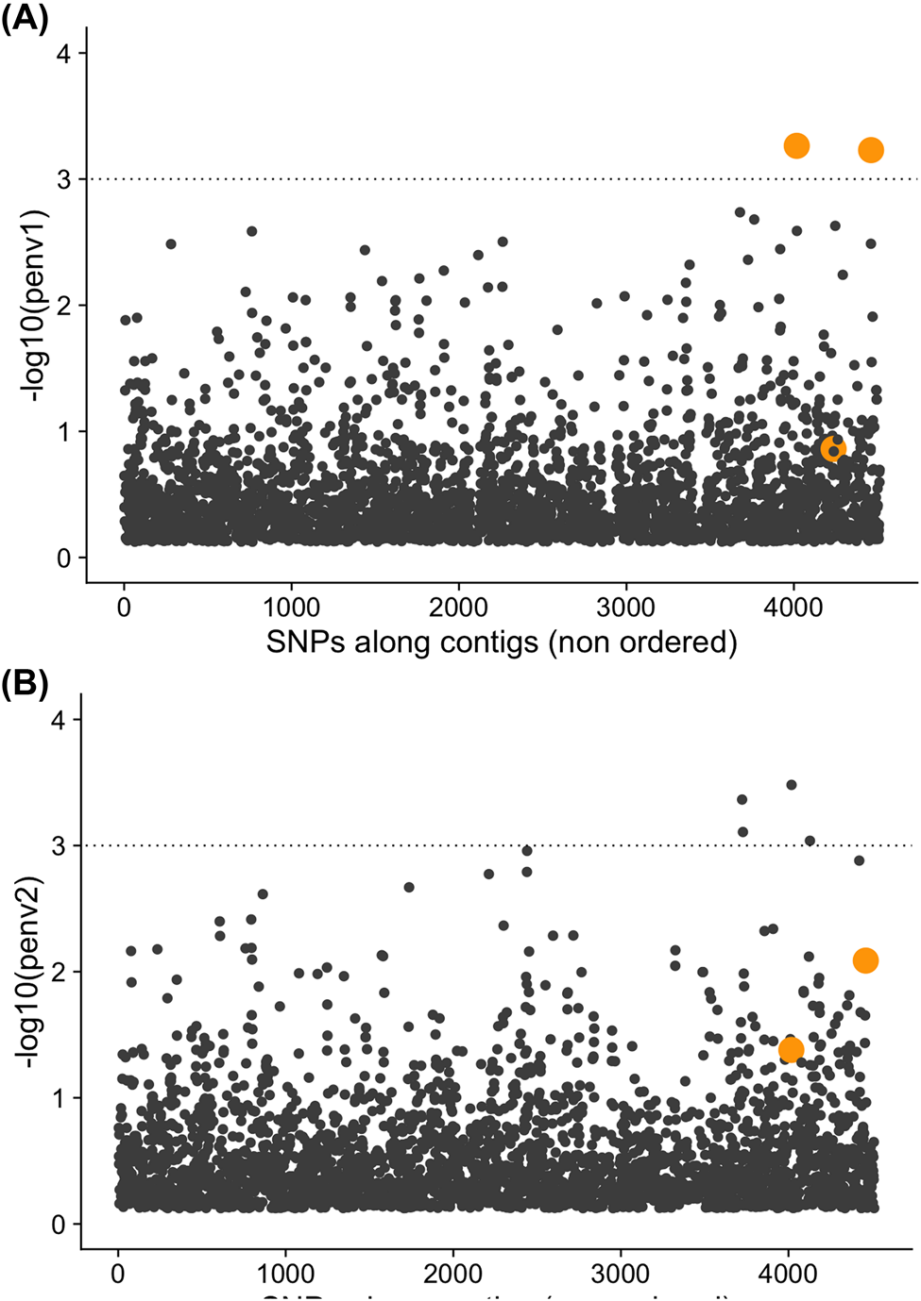
SNP‐environment associations. Manhattan plots depict –log10(*P*‐value) for tests for the SNP‐EnvPC1 (A) and SNP‐EnvPC2 (B) associations. Orange dots denote the three SNPs in Table 2, Panel A used to build the genotype to ecotype map (Figure 3A). For graphical convenience, only *P*‐values <0.75 are depicted (so in Figure 4B, one of the orange dots is not displayed).

As ecotypes, and chemotypes nested within ecotypes, track environmental variation, we expect genetic variants underlying this phenotypic variation to be associated with the environmental variation. We thus examined the extent to which SNPs were associated with environmental variation. The SNP‐environment covariation measures the efficacy of ongoing selection to segregate alleles spatially despite the homogenizing effect of gene flow. To do so, we tested whether SNPs frequencies covary with the PC1 and PC2 axes of environmental variation that account for 45% and 28%, respectively, of the total abiotic environmental variation we measured (Figure S1). Using a logistic regression framework that accounts for local genetic differences, we found only a few SNPs with suggestive SNP‐environment associations (Figure 4), but none of these are statistically significant after correction for multiple testing across all SNPs.

Among the six major SNPs determining eco‐ and chemotype identity (Table 2; Figure 3A,B), the three SNPs that explain ecotype identity are those with the strongest changes in allele frequency across environment (Figure S2; Table S3). These SNPs (Contig71000, and especially Contig12377 and KR920616.1; Table 2) show associations with the abiotic environmental variation captured by PC1 axis of environmental variation. This is expected given that the frequency of the two phenolic chemotypes, carvacrol and to a lesser extent thymol, covary with the PC1 axis: phenolic types are rare in populations located at sites with high values of environmental PC1 (Figure 2C), which corresponds to harsh winters (Figure S1).

Our study relies on environmental variation at 21 sites and lacks statistical power to detect weaker SNP‐environment associations. It may further be argued that some SNP‐chemotype associations could be caused by population structure not accounted for by the five SNP PCs. However, these associations still hold even when accounting for population structure by using up to 10 SNP PCs (Figure S3). Moreover, if such associations were a mere artifact of population structure (Figure S4), we would not expect SNPs that are associated with particular ecotypes or chemotypes to be located in genes of the monoterpene pathway, as invariably found in our study.

In contrast to results for ecotypes, the frequency of SNPs displaying strong associations with chemotype identity had much weaker associations with environmental variables. Theory predicts that the effect of selection on a locus controlling phenotypic variation for a fitness related trait can be influenced by selection jointly acting on other loci (Chevin and Hospital 2008; Chevin 2019), and that redundancy has a profound impact on the evolution of polymorphisms (Láruson et al. 2020). The amount of segregating genotypic redundancy controls how phenotypic selection will leave (or not) detectable footprints at the genome level. In our study system, ecotypes are determined by few loci and low segregating genotypic redundancy (Figure 3A), whereas chemotype determination involves more loci and more variable segregating genotypic redundancy (Figure 3B). The consequence of this is that genetic variation at the loci contributing to ecotypic variation more closely aligns with environmental variation, and the signal is much weaker at the loci that account for a comparatively smaller fraction of chemotype variation. In addition to affecting how phenotypic selection may leave a detectable footprint at the genomic level, genotypic redundancy may also provide robustness to swamping by migrating alleles. This may contribute to maintain the high number and spatial distribution of chemotypes despite the high gene flow observed in our study region.

## Conclusion and Perspectives

We demonstrate in this study how ecotypic differentiation can be maintained in the face of considerable gene flow. Ecotype identity is under oligogenic control and variation at a single gene explains the majority of ecotype variation. This has consequences for how selection operates on phenotypic variation to maintain adaptive differentiation. Loci accounting for most of the heritable variation in a fitness‐related trait undergo the strongest selection at the genotype level (Chevin and Hospital 2008; Chevin 2019). Accordingly, allele frequencies of SNPs located in the three genes for ecotype differentiation also covary with the environmental variation (env PC1) that is tracked by ecotypes. The finding of major effect genes, combined with low genotype to ecotype segregating redundancy, can explain the rapid increase in the frequency of the phenolic ecotype documented in the same study region over the last 50 years and hypothesized to be driven by recent climatic warming (Thompson et al. 2013).

The chemotypes that we studied are also found in many other species of *Thymus* and other *Lamiaceae* genera. The ecological roles of monoterpenes are manifold and include deterrence of herbivores (Linhart and Thompson 1999; Gershenzon and Dudareva 2007; Ehlers et al. 2020), allelopathy (Gershenzon and Dudareva 2007; Ehlers et al. 2014), and protection against abiotic stress (Possel and Loreto 2013). Thyme is also a “community engineer”: leaching of chemicals from leaves modifies the local soil environment and in turn the neighboring plant community, and these effects vary with the identity of the monoterpene (Ehlers and Thompson 2004; Grøndahl and Ehlers 2008; Ehlers et al. 2016). Unravelling the genetics of this variation is a first step toward understanding the origin and maintenance of this key polymorphism that has ecologically important effects on community dynamics.

## Supporting information


**Fig. S1**. A: Projection of individual environmental variables on principal components of environmental variation. B: Correlation between PC1 and the minimum temperature registered in January. C: Correlation between PC2 and the number of days with temperature exceeding 40 degrees C.
**Fig. S2**. SNPs frequency clines along environment gradient at 6 SNPs explaining ecotype and chemotype identity. Environmental variation is measured using the first principal component of environmental variation.
**Fig. S3**. Robustness of PCs correction for population structure when detecting associations.
**Fig. S4**. SNPs PCs colored by site of origin of individuals.
**Table S1**. Repeatability of SNPs genotyping
**Table S2**. sample size, Fis and Fst at the top 6 candidate loci
**Table S3**. Effect of environment on change in allele frequency at 6 SNPs with the strongest associations to ecotype and chemotype identity.
**Table S4** Model comparison for genotype‐ ecotype associations using 5 or 10 PCs.
