# Supplementary material for: From genotype to phenotype: Genetic redundancy and the maintenance of an adaptive polymorphism in the context of high gene flow
Source: Evol Lett. 2022 Feb 22;6(2):189–202. doi: 10.1002/evl3.277 (PMC8966474; doi:10.1002/evl3.277)
Supplement: Supplementary file 1 — Fig. S1. A: Projection of individual environmental variables on principal components of environmental variation. B: Correlation between PC1 and the minimum temperature registered in January. C: Correlation between PC2 and the number of days with temperature exceeding 40 degrees C. Fig. S2. SNPs frequency clines along environment gradient at 6 SNPs explaining ecotype and chemotype identity. Environmental variation is measured using the first principal component of environmental variation. Fig. S3. Robustness of PCs correction for population structure when detecting associations. Fig. S4. SNPs PCs colored by site of origin of individuals. Table S1. Repeatability of SNPs genotyping Table S2. sample size, Fis and Fst at the top 6 candidate loci Table S3. Effect of environment on change in allele frequency at 6 SNPs with the strongest associations to ecotype and chemotype identity. Table S4 Model comparison for genotype‐ ecotype associations using 5 or 10 PCs. [file EVL3-6-189-s001.docx]

**Supplementary Information**

Corresponding author: Thomas Bataillon

Email: [tbata@birc.au.dk](mailto:tbata@birc.au.dk)

**This PDF file includes:**

Figures S1 to S4

Tables S1 to S3

Supplementary text for methods:

reproducibility of SNP calls and genotypes

details of statistical analysis

locus targeted sequencing protocols

SI References


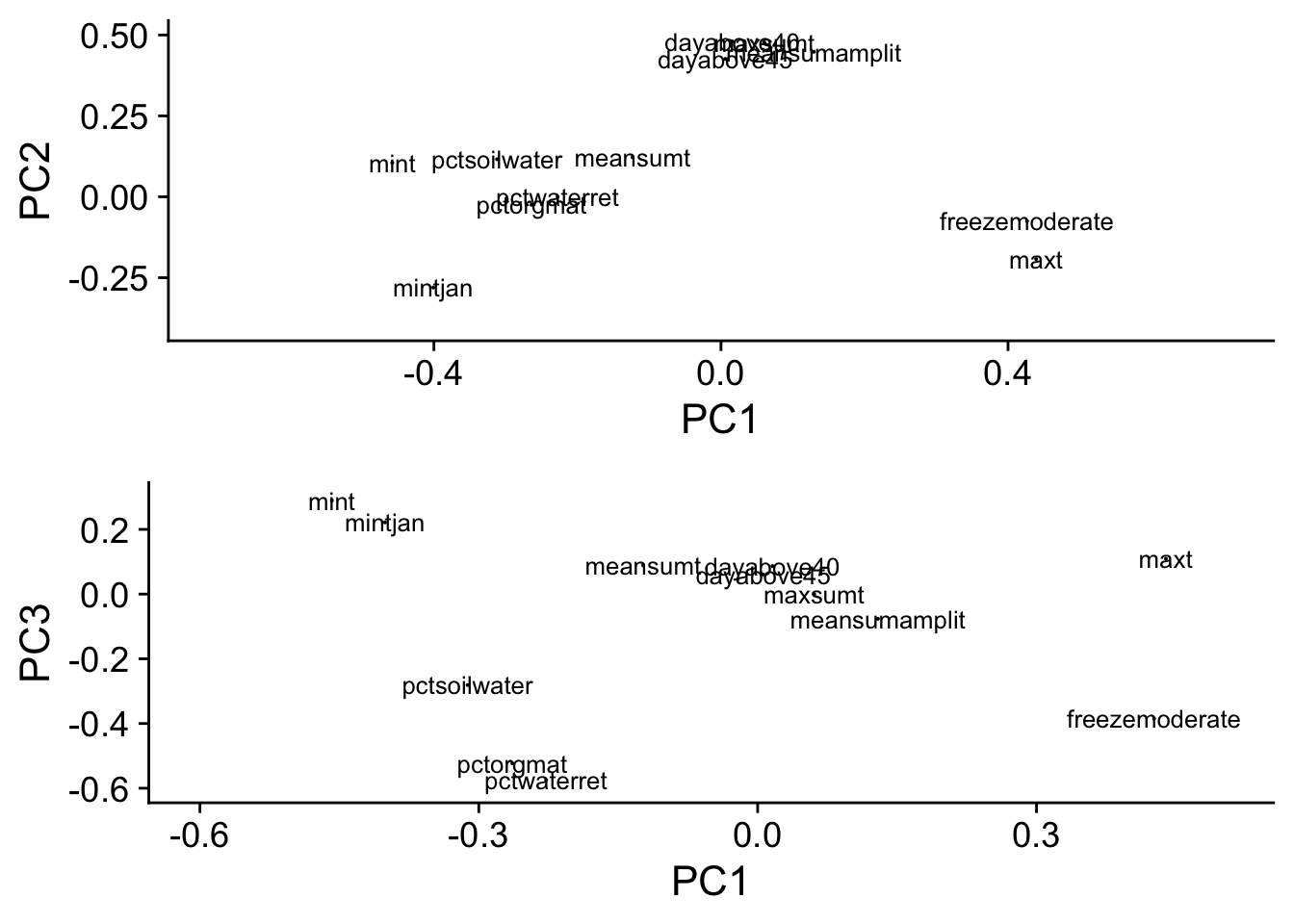


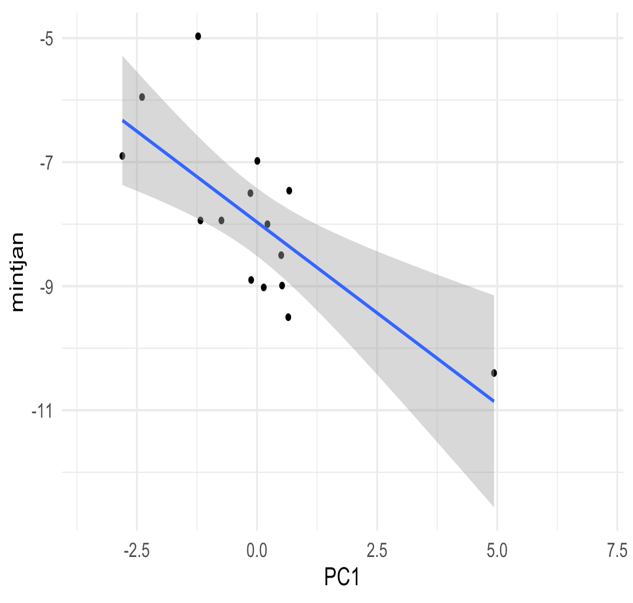

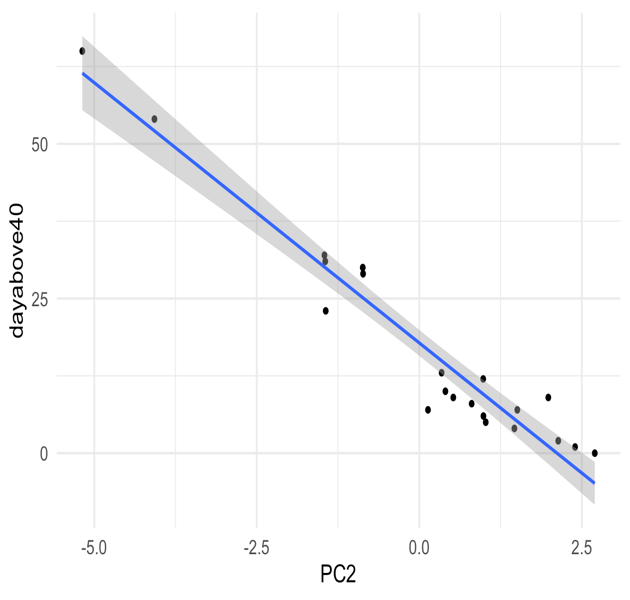


Fig. S1.

A: Projection of individual environmental variables on principal components of environmental variation.PC1, 2 and 3 account for 45%, 28% and 12% of the total environmental variation measured across sites. The individual variables used (centered and reduced to unit variance) were percentage water in soil, soil water retention and percentage organic matter in the soil ("pctsoilwater" "pctwaterret" "pctorgmat"), the mean daily minimum and maximum temperature in winter ("mint" "maxt"), the minimum temperature in the coldest month ("mintjan"), the mean daily minimum and maximum temperature the summer ("maxsumt" "meansumt"), the number of days where moderate to strong freezing was recorded (below -8C) "freezemoderate", the number of days exceeding 40C or 45C ("dayabove45" "dayabove40"), and the mean summer daily temperature amplitude ("meansumamplit"). Note that several of these individual variables are highly correlated as illustrated by their overlap in PCs plots.

B Correlation between PC1 and the minimum temperature registered in January.

C. Correlation between PC2 and the number of days with temperature exceeding 40 degrees C.

Fig. S2.

SNPs frequency clines along environment gradient at 6 SNPs explaining ecotype and chemotype identity. Environmental variation is measured using the first principal component of environmental variation. See supplementary Table 4 for R^2^ and p-values associated with each model.


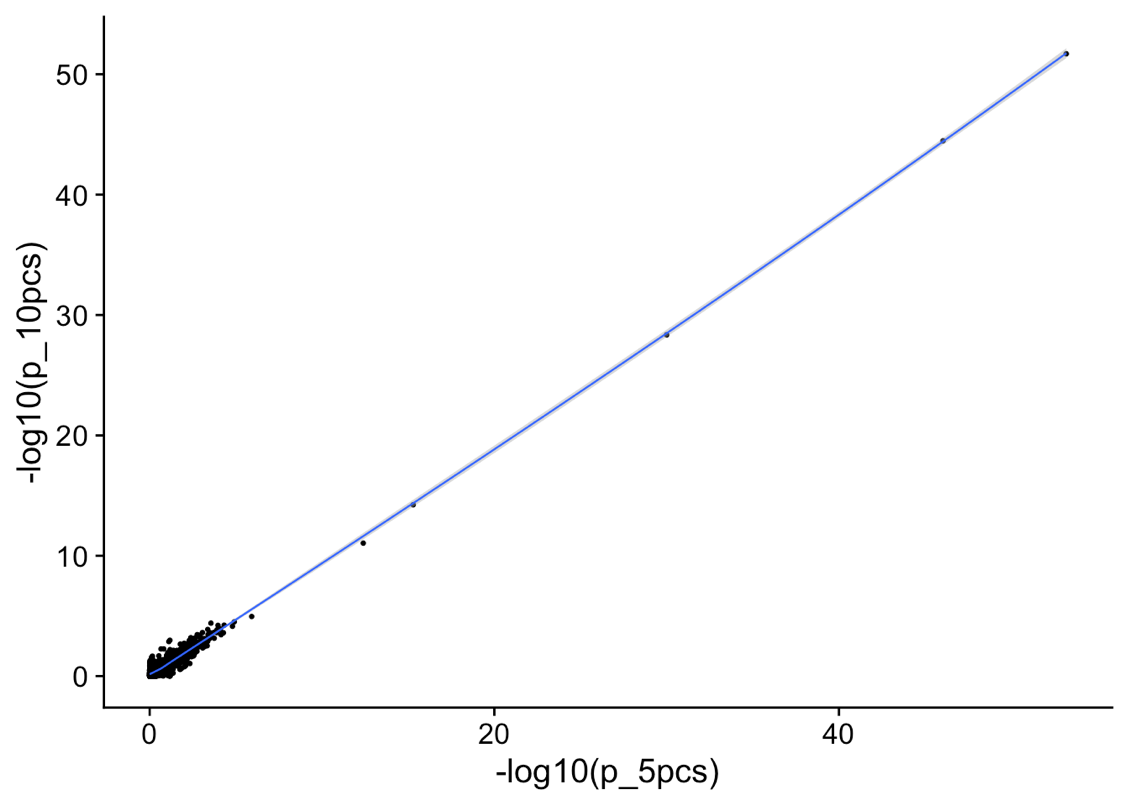


Fig. S3. Robustness of PCs correction for population structure when detecting associations.

We plot the correlation between -log10(p-values) for ecotype-SNP associations when using either 5 (p_5pcs) or 10 PCs (p_10PCs) of SNP variation to correct for background population structure. Clearly there is a very strong correlation between both p-values. The blue lines indicate a “loess” (local non-parametric) regression. Each dot indicates an individual SNP.

**
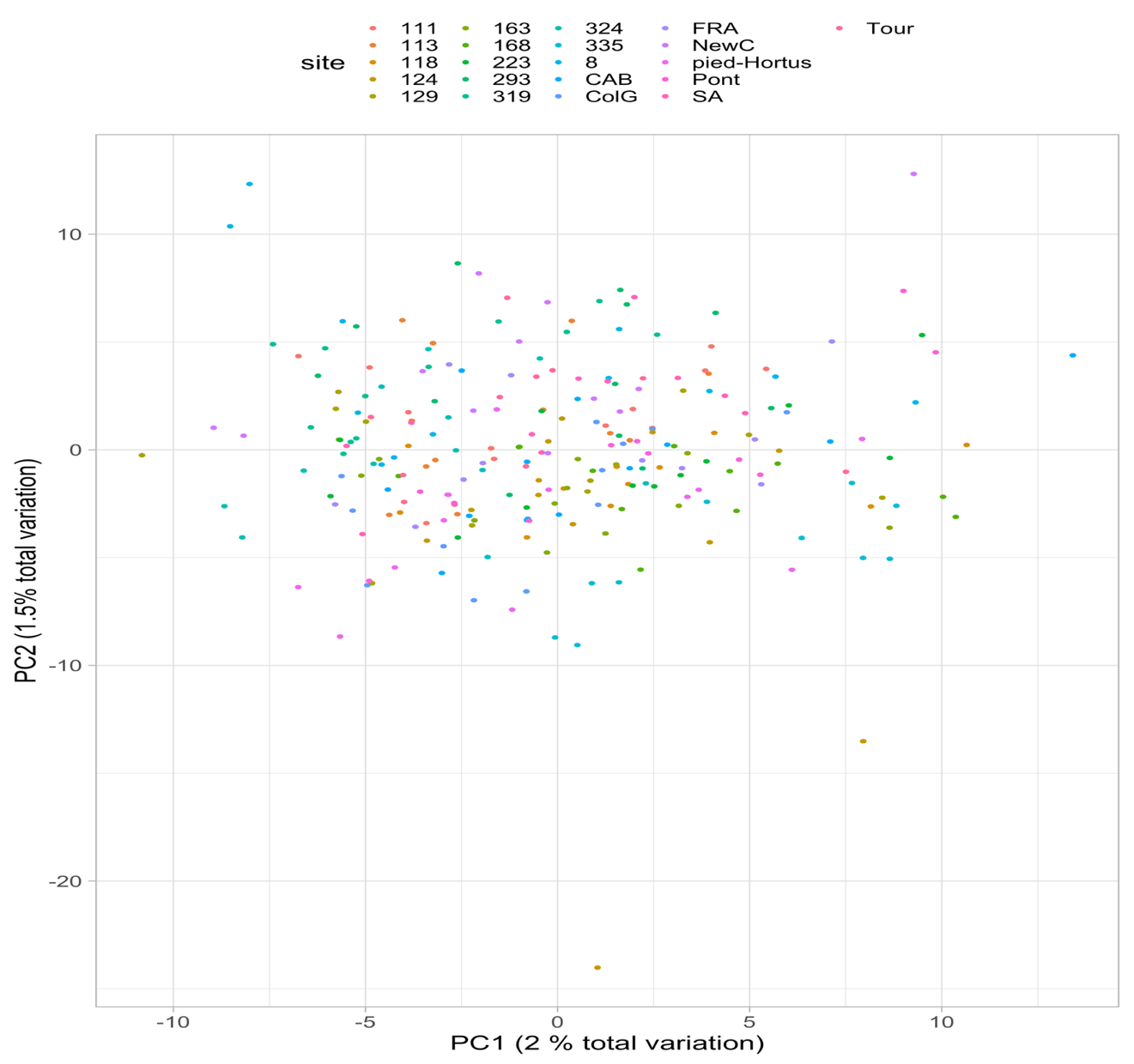
**

Fig. S4. SNPs PCs colored by site of origin of individuals.

Display is identical as in Figure 1B. But, here we color code every individual by location of sampling -instead of ecotype identity- to illustrate that there is no clustering of samples that are spatially close (belonging to the same site).

Table S1. Repeatability of SNPs genotyping

| Principal Component | Repeatability^$^ | Cumulative R^2^ |
| --- | --- | --- |
| PC1 | 0.998 | 0.020 |
| PC2 | 0.999 | 0.035 |
| PC3 | 0.996 | 0.049 |
| PC4 | 0.995 | 0.062 |
| PC5 | 0.999 | 0.076 |
| PC6 | 0.998 | 0.088 |
| PC7 | 0.995 | 0.100 |
| PC8 | 0.996 | 0.111 |
| PC9 | 0.990 | 0.123 |
| PC10 | 0.992 | 0.134 |

$ Repeatability is calculated by Pearson's product-moment correlation coefficient of each PC coordinate using the coordinates of 23 independent paired technical replicates.

Cumulative R^2^ indicates the proportion of the total variance explained by PC1, PC1+ PC2, etc.

Table S2. sample size, Fis and Fst at the top 6 candidate loci

| Gene / snp | n | ref | alt | freq_alt | Fis | Fst |
| --- | --- | --- | --- | --- | --- | --- |
| Contig71000_648 | 250 | T | C | 0.75 | 0.00 | 0.0227 |
| Contig12377_126 | 200 | A | T | 0.15 | 0.00 | 0.227 |
| KR920616.1_174 | 240 | A | T | 0.14 | 0.00 | 0.184 |
| JX946358.1_1452 | 190 | T | C | 0.17 | 0.00 | 0.065 |
| KC461937.1_1279 | 240 | G | A | 0.2 | 0.00 | 0.012 |
| KM272332.1_359 | 240 | T | A | 0.11 | 0.09 | 0.252 |

n: number of individuals genotyped

ref and alt denote the two SNP alleles.

Fis denotes the deficit (when negayive) or excess of homozygotes relative to Hardy-Weinberg proportions and is estimated along with Fst using Weir and Cockerham’s estimators to account for the effect of population structure.

Table S3: Effect of environment on change in allele frequency at 6 SNPs with the strongest associations to ecotype and chemotype identity.

| Gene / SNP position | Beta1 | R2 env1 | p1 | Beta2 | R2 env2 | p2 |
| --- | --- | --- | --- | --- | --- | --- |
| Contig71000 / 648 | -1.475 | 0.182 | 0.138 | -0.123 | 0.001 | 0.902 |
| Contig12377 / 126 | 3.476 | 0.161 | 0.001 | 2.000 | 0.056 | 0.042 |
| KR920616.1 / 174 | 3.452 | 0.189 | 0.001 | -2.655 | 0.112 | 0.008 |
| JX946358.1 / 1452 | 0.875 | 0.018 | 0.386 | -1.845 | 0.078 | 0.068 |
| KC461937.1 / 1279 | 1.079 | 0.113 | 0.287 | 2.190 | 0.532 | 0.021 |
| KM272332.1 /359 | -1.412 | 0.040 | 0.142 | 2.143 | 0.098 | 0.022 |

Beta1 (respectively Beta2) is the standardized slope (Beta = slope /SE(slope) measuring the effect of environmental variation (respectively envPC1 and envPC2) on SNP allele frequency (as fitted by a logistic regression, see methods).

R2 env1 (respectively R2 env2) measures the portion of variance in allele frequency explained the model relative to a background model without the envPC1 (envPC2). R2 are obtained from ratio of deviance of logistic regression models (with and without the envPCs as predictor, see methods)

p1 (respectively p2) is the p-value for the likelihood ratio test of association of a focal SNP with envPC1 (respectively envPC2). P-values in bold are those <0.05/(2x6) = 0.00417.

Table S4: Model comparison for genotype- ecotype associations using 5 or 10 PCs.

| Gene / SNP position | *P*_5_ | *P*_10_ | AIC_5_ | AIC_10_ |
| --- | --- | --- | --- | --- |
| Contig71000 / 648 | 6.22e-54 | 1.89e-52 | 81.1 | 85.4 |
| Contig12377 / 126 | 9.82e-31 | 4.27e-29 | 178 | 183 |
| KR920616.1 / 174 | 3.98e-13 | 8.71e-12 | 263 | 266 |

*P*_5_ and *P*_10_ are the p-value for genotype-ecotype association obtained by a likelihood ratio test comparing logistic models M0 and M1 to detect snp- ecotype association including respectively 5 and 10 principal components of SNP variation for Model M0 and M1. AIC_5_ and AIC_10_ are the Akaike information criteria of each model M1 using 5 and 10 principal components of SNP variation for Model M1 (see SNPs-Ecotype and SNPs-Chemotype association method section for further details on model fitting).

Dataset S1 (separate file). Capture Adapters.xlsx.

Excel file containing the sequence of the specific hexamer barcodes used for genomic capture (see supplementary text below)

**Supplementary methods: reproducibility of SNP calls and genotypes**

## SNP quality control and SNPs genotyping

To minimize instances of collapsed paralogs, we only called SNPs in regions targeted by enrichment before sequencing A similar protocol used in the context of allopolyploid genomes such as durum wheat) proved to be a reliable strategy.

We only used SNPs that were detected by both SNP calling methods (GATK and read2snps) and we also restricted our attention to SNPs with a minor allele frequency equal or greater to 0.1 (averaged over all individuals). The method read2snps is also designed for SNP calling in the re-sequencing of individuals from non-model species in mind and can detect instances of SNPs created by un-collapsed paralogs that have abnormally low Fis (no instances detected among the set of SNPS we use here). This yielded 4520 commons SNPs. The minor allele frequency threshold was chosen to ensure both reliability of SNPs genotypes but also to have minimum power for detecting association between a SNP genotype and chemotype (or environmental) variation. We also recorded the GATK quality scores, Q. Q scores can be used as proxy for expected error rates in the absence of true technical replicates (but see below). Quality scores for the set of 4520 common SNPs was high (meanQ =19.95, SE = 0.07), yielding an expected mean error rate of 1%.

**Repeatability of SNP genotypes**

Instead of solely relying on GATK quality scores, we used twenty-three independent technical replicates (i.e. individual plants with the same DNA extraction but completely independent myBAITS enrichment, library preparation, sequencing, read mapping and variant calling) to assess the overall reliability of our sequencing and SNP calling piepline.

We first computed the proportion of the time the exact same genotype was called (perfect genotype recall) across the technical replicates. We found that common SNPs (minor allele frequency of 0.1 or higher) were highly reproducible (mean proportion of perfect recalls: 0.996, median of 1). As a complementary check, we computed the correlation of scores on the first 10 PCA coordinates for the replicates. Doing so confirms the overall reproducibility of our sequencing, SNP calling and SNP genotyping on the set of common SNPs (Table S1). Last, we checked that the proportion of perfect genotype recalls for the 6 SNPs displaying the highest SNP-chemotype associations (Table **S**2) and also confirmed a high reproducibility for these SNPs (109 perfect genotype recalls out of 110 genotypes called).

**Supplementary methods: details of statistical analysis**

**SNPs-Ecotype and SNPs-Chemotype association**

We used a logistic regression framework where we are evaluating association between a SNP genotype and a binary phenotype (here a given chemotype or ecotype identity) by comparing the fit of two models:

A background model (hereafter M0) where 5 PCs of common SNPs variation are used to predict chemotype:

M0 binary phenotype ~ PC1 + PC2 + PC3 + PC4 + PC5

We expect M0 to have some predictive power because of the genetic differentiation between populations and the fact that populations differ on average for their ecotype (chemotype) composition (figure S2).

A SNP model (hereafter M1) that also uses a single SNP genotype (coded as 0, 1, 2) as predictor

M1 binary chemotype ~ PC1 + PC2 + PC3 + PC4 + PC5 + SNP

M1 models were fitted by maximum likelihood for each common SNP. This was done using the *glm*() function in R and model M1 was implemented using a logistic link and a binomial distribution for the binary phenotype.

The phenotypic effect associated with an individual SNP was computed using the (pseudo) R^2^ associated with model M1 for this SNP. As M0 and M1 are generalized linear models, several ways to compute R^2^ have been proposed and there is no agreement on an “optimal” analog to R^2^ measures. We computed R^2^ as 1 – Dev(M1)/Dev(M0), where Dev(Mi) is the deviance of model Mi. In brief the rationale for this choice is as follows. Deviance of M0 is analogous to a residual sum of square in a linear regression model: the bigger the lack of fit the bigger Dev(M0). Accordingly, deviance of M1 measures the lack of fit of model M1. This measures the improvement in our ability to fit individual (binary) phenotypic values across individual when we use the extra genotype information brought by a single SNP.

To test for association at each SNP and obtain a formal p-value for association, we used a likelihood ratio test and used twice the difference in deviance between M0 and M1 (Gobs = 2 (ln M1 – ln M0)) as test statistic. We assumed that under the assumption that data comes from model M0, we expect Gobs to be chi-squared distributed with one degree of freedom. Using the background model M0 as null model guards against spurious SNP-chemotype association merely due to the fact that there is some (weak) genetic differentiation between populations and differences in chemotype/ecotype frequencies across populations.

Just as in the calculation of genetic differentiation (Fst), we used 3920 SNPs, where the minor allele frequency was above 0.1, and all individuals that were both genotyped and phenotyped (n=248 after quality control on SNPs genotyping) to detect associations.

We used a strict Bonferroni correction to account for the fact that associations between SNP and the binary ecotype and chemotype were tested at numerous SNPs. Here, given that nT= 3920 SNPs were tested, α/nT is used as significance cutoff for each individual SNP. Setting α = 0.05, this amounts to require an individual SNPs significance threshold 0.05/nT= 1.27551e-05.

Visuals checks were performed on the empirical distribution of p-values for each phenotype to ensure that these were properly calibrated. We expect most p-values to conform to M0 and accordingly a uniform distribution in [0,1] for p-values with a minor bulge of lower p-values (for SNPs displaying associations as specified underM1).

We checked the robustness of our approach by recomputing SNP- ecotype associations using 10 PCs of SNPs variation (instead of 5) in implementing M0 and M1. The rationale for doing so is to validate that our modelling approach is robust to the choice of the number of PCs included in M0 to account for population structure. Model comparison revealed that implementing models using 10 PCs instead of 5PCs do not overall provide significantly better fit of the data, and model fit was even worse for the top SNPs in ecotype-SNP associations (Table S4). Moreover, computing a genomic factor to quantify potential inflation of the LRT statistic, because of possible unaccounted population structure, revealed no improvement by using 10 Pcs over 5 PCs (both inflation factors were very close to 1: 1.11 and 1.15 respectively with 5 and 10 PCs). We checked that SNPs scores (defined as -log10(p-values)) computed either method are highly correlated (observed correlation 0.98, p-value < 2.2 10^-16^). We also checked that top p-value ranking was insensitive to the modelling choice of 5 vs 10 PCs (Figure S3). Given these facts, we felt compelled to use 5 PCs throughout both for SNPs- phenotypes and SNPs- Environment associations.

**Details of methods for testing for SNPs- environment association**

A background model (M0) where 5 PCs of common SNPs variation are used to predict allele frequency variation at a focal SNP:

M0: SNP ~ PC1 + PC2 + PC3 + PC4 + PC5

M0 is compared to an “environmental gradient model” that uses an environmental covariate (coded as a continuous variable) as supplementary predictor of the SNP frequency.

M1 SNP ~ PC1 + PC2 + PC3 + PC4 + PC5 + ENV

A likelihood ratio test comparing M0 and M1 test is used as described above to test for SNP- ENV association and obtain a p value significance of the association. A pseudo R^2^ measure is also be computed as described above, and the slope associated to the ENV variable. To limit the number of tests, we used the first two PCs of environmental variation as ENV predictor. These two PCs account for a substantial fraction of the site to site environmental variation measured, and we know that ecotype (and chemotypes) co vary with these PCs.

Using the background model M0 with 5 PCs as null model guards against spurious SNP-ENV association due to genetic differentiation between sites and differences of local environment across sites. We fitted (M0, M1) models using each common SNP as response variable and using separately PC1 and PC2 of environmental variation as ENV covariate.

All generalized models described above were using multiple predicting variables (such as PC of SNPs and PCs of environmental variation as well as individual SNPs genotypes) and were checked for correlation between predictors by computing variance inflation factors [4] implemented in the *car::vif*() function R package *car* version 3.0-8 [5]*.*

**Supplementary method: locus targeted sequencing protocols**

**Plant DNA purification**

DNA was extracted from 15 mg of fresh young leaves with the Chemagic DNA Plant Kit (Perkin Elmer Chemagen, Baesweller, DE, Part # CMG-194), according to the manufacturer’s instructions. The protocol is adapted to the use of the KingFisher Flex™ (Thermo Fisher Scientific, Waltham, MA, USA) automated DNA purification workstation.

**Construction of enriched library and sequencing**

Genomic library preparation for multiplexed individuals and enrichment step by capture follow published protocols (1,2) with some modifications. The baits where designed bioinformatically to target a set of candidate genes identified by transcriptome sequencing (3) as well as candidate for known genes encoding enzymes of the monoterpene biosynthesis pathway previously identified in T vulgaris.

**A Target preparation, construction of barcoded genomic libraries**

1 : For each individual, 1 µg of total DNA (in 100 µL of water) are sheared using a Bioruptor Pico (Diagenode, Seraing, BE) sonication device in 500 µl microtubes to a targeted 300 bp DNA fragment size using parameters of the 300pb standard protocol for DNA shearing. *(*<https://www.diagenode.com/files/protocols/Standard_protocols_for_DNAShearing.pdf>*).*

2: 400 ng of fragmented DNA (in 40 µl of water) are blunted and 5’ phosphorylated using the Thermo Scientific Fast DNA End Repair Kit (Thermo Fischer Scientific, Waltham, MA, USA, Part # K0771). A clean-up step is performed with 1 x volume of Agencourt AMPure XP magnetic beads. The elution volume is 20 µL.

3: Fragmented and repaired DNA are individually controlled (sizing and estimation of the concentration) by electrophoresis on a AATI Fragment Analyzer™ (Advanced Analytical Technologies, Ankeny, IA, USA) device with the DNF-474 High Sensitivity Fragment Analysis Kit.

4: 50 ng of fragmented DNA are ligated with 4 pmol of PE-P5 and MPE-P7 adapters. Each PE-P5 and each MPE-P7 adapter carries the same specific hexamer barcode (2), see file Capture Adapters.xls in Dataset S1) Reactions are conducted in 15 µl final volume with 1 unit of T4 DNA ligase for 1 hour at 22 °C followed by a heat inactivation step at 65°C for 10 minutes.

5: 48 samples (corresponding to 48 hexamer barcodes on the PE-P5 and PE-P7 adapter) are pooled. A clean-up step is performed with 1.8x volume of Agencourt AMPure XP magnetic beads. The elution volume is 94µL.

6: A nick fill-in step is performed using 64 units of Bst DNA polymerase (New England Biolabs, Ipswich, MA, USA, Part # M0275), 1x ThermoPol® reaction buffer, 250 µM dNTP in 120 µl final volume and incubated for 15 minutes at 37°C. A clean-up step is performed with 1.8x volume of Agencourt AMPure XP magnetic beads. The elution volume is 40 µL.

7: For each pool of 48 samples, a pre-hybridization PCR is performed using the Phusion® High-Fidelity PCR Master Mix (Thermo Fischer Scientific, Part # 1040-2678) with 200 nM PreHyb-PE_F (CTTTCCCTACACGACGCTCTTC) and 200 nM PreHyb-MPE_R (TGACTGGAGTTCAGACGTGTG) primers in a final volume of 100µl.

Thermocycling parameters: 3 minutes at 98°C, followed by 12 cycles of 80 seconds at 98°C; 45 seconds at 55°C and 60 seconds at 68°C, with a final elongation of 10 minutes at 72°C. A clean-up step is performed with 1.8x volume Agencourt AMPure XP magnetic beads. The elution volume is 20 µL.

**B Enrichment, capture by hybridisation**

The protocol used is based on Mascher et al (2), the User Manual of the MYBaits Sequence Enrichment for Targeted Sequencing kit (<http://www.mycroarray.com/pdf/MYbaits-manual-v2.pdf>) and on the Roche NimbleGen SeqCap EZ Library SR User’s Guide (<http://sequencing.roche.com/products/nimblegen-seqcap-target-enrichment/seqcap-reagents.html>**)**

**B-1 First round of hybridization of the barcoded libraries to biotinylated RNA probes**

8: Prior to hybridization, 10 μl of Roche Diagnostics (Indianapolis, IN, USA) proprietary SeqCap EZ Developer Reagent (Roche, Part # 06684335001) were added to a 1.5-ml tube containing 0,5 μg of the 48 barcoded samples genomic library.

Next were added as blocking oligos:

- 1 µl (100 pmol/µl solution) of the P5 adapter blocking oligo , 5’-AGATCGGAAGAGCGTCGTGTAGGGAAAG
- and 1 µl (100 pmol/µl solution) of the MP7 adapter blocking oligo, 5’-AGATCGGAAGAGCACACGTCTGAACTCCAGTCA,

designed to block the truncated segment of TruSeq DNA library adapters during the sequence capture.

The mixture was dried down in a SpeedVac at 43°C during 20 to 30 min.

9: 7.5 μl of 2 × Sequence Capture Hybridization Buffer (tube 5, SeqCap EZ Hybridization and Wash Kit, Roche, Part # 05634261001) and 3 μl of Hybridization Component A (tube 6, SeqCap EZ Hybridization and Wash Kit, Roche, Part # 05634261001) were added. The hybridization cocktail was vortexed for 10 sec and collected by centrifugation. Following denaturation in a heat block (95°C, 10 min) the sample was transferred to a 0.2 ml PCR tube containing 80 ng of biotinylated RNA probes (4.5 µL of MYBaits Capture Probe).

10: The hybridization sample (15 μl) was incubated in a thermocycler (lid heated to 57°C) at 47°C for 64 h.

**B-2 First round of washing of the captured library.**

11: Streptavidin coupled magnetic beads are previously equilibrate as recommended by the Roche-Nimblegen protocol. Invitrogen Dynabeads MyOne™ Streptavidin C1 (Invitrogen, Thermo Fischer Scientific, Part # 65001) at 10 µg/µl were thoroughly vortexed, aliquoted (50 μl per hybridization) into 1.5-ml tubes and prepared for the affinity purification of captured DNA. The tubes were placed in a DynaMag-2 magnet (Invitrogen, Part # 123-21D) for 2 min. The clear liquid was discarded and 100 μl of 1 X Bead Wash Buffer (Tube 7, SeqCap EZ Hybridization and Wash Kit, Roche, Part # 05634261001) were added. Tubes were vortexed, placed back in the magnet, the clear liquid was removed, and the washing was repeated once. Dynabeads were resuspended in 50 μl 1 x Bead Wash Buffer, transferred into PCR plates and collected using a Agencourt SPRIPlate 96R (Agencourt, Part # A32782) . The clear supernatant was discarded.

12: The hybridization sample was added to the wet Dynabeads and mixed thoroughly by pipetting up and down. Using a thermocycler (lid heated to 57°C) at 47°C for 45 min the captured sample was bound to the Dynabeads. The sample was vortexed for 3 sec in 15-min intervals to ensure that the Dynabeads remain in suspension. Dynabeads plus bound DNA (15 μl) were washed by adding 100 μl 1 X Wash Buffer 1 (pre-heated to 47°C for 1 h) (Tube 1, SeqCap EZ Hybridization and Wash Kit, Roche, Part # 05634261001) and vortexing for 10 sec.

13: The suspension was transferred to a 1.5-ml tube and placed in a DynaMag-2 device, and the supernatant was discarded once clear. Washing was continued by adding 200 μl 1 X Stringent Wash Buffer (pre-heated to 47°C for 1 h) (Tube 4, SeqCap EZ Hybridization and Wash Kit, Roche, Part # 05634261001) The sample was mixed by pipetting avoiding a major temperature drop and incubated for 5 min at 47°C. The tube was placed in the DynaMag-2 magnet, the liquid was discarded and the washing at 47°C with 1 X Stringent Wash Buffer was repeated once.

14: 200 μl 1 X Wash Buffer 1 (pre-heated to room temperature) was added to the Dynabeads plus bound DNA. The sample was vortexed for 2 min and the liquid was collected to the tube's bottom. Following magnetic concentration the liquid was discarded, and the sample was washed at room temperature with 200 μl 1 X Wash Buffer 2 (vortexing for 1 min) (Tube 2, SeqCap EZ Hybridization and Wash Kit, Roche, Part # 05634261001), followed by a wash with 200 μl 1 X Wash Buffer 3 (vortexing for 30 sec) (Tube 3, SeqCap EZ Hybridization and Wash Kit, Roche, Part # 05634261001) as described for washing with Wash Buffer 1. The tube was removed from the magnet, the bead-bound captured library was resuspended in 25 μl PCR-grade water and the entire sample (beads + liquid) was transferred to a 0.2 ml PCR tube.

**B-3 Second round of hybridization of the barcoded libraries to biotinylated RNA probes**

15: The captured sample (25 μl) was denatured by incubation in a thermocycler (lid heated to 105°C) at 95°C for 3 min. 21 µl of the denatured solution were quickly transferred on to a 1,5 ml microtube.

16: Next were added to the captured sample

- 1 µl (10 pmol/µl solution) of the P5 adapter blocking oligo
- 1 µl (10 pmol/µl solution) of the MP7 adapter blocking oligo,
- 1 μl of SeqCap EZ Developer Reagent.

The mixture was dried down in a SpeedVac at 43°C during 10 to 20 min.

17: 7.5 μl of 2 × Sequence Capture Hybridization Buffer (tube 5, SeqCap EZ Hybridization and Wash Kit) and 3 μl of Hybridization Component A (tube 6, SeqCap EZ Hybridization and Wash Kit) were added. The hybridization cocktail was vortexed for 10 sec and collected by centrifugation. Following denaturation in a heat block (95°C, 10 min) the sample was transferred to a 0.2 ml PCR tube containing 15 ng of biotinylated RNA probes (1 µL of MYBaits Capture Probe) and 3,5 µl of UP water.

18: The hybridization sample (15 μl) was incubated in a thermocycler (lid heated to 57°C) at 47°C for 20 h.

**B-2 Second round of washing of the captured library.**

19: Streptavidin coupled magnetic beads are prepared as previously described (#11)

20: The hybridization sample was added to the wet Dynabeads and mixed thoroughly by pipetting up and down. Using a thermocycler (lid heated to 57°C) at 47°C for 45 min the captured sample was bound to the Dynabeads. The sample was vortexed for 3 sec in 15-min intervals to ensure that the Dynabeads remain in suspension. Dynabeads plus bound DNA (15 μl) were washed by adding 100 μl 1 X Wash Buffer 1 (pre-heated to 47°C for 1 h) (Tube 1, SeqCap EZ Hybridization and Wash Kit) and vortexing for 10 sec.

21: The suspension was transferred to a 1.5-ml tube and placed in a DynaMag-2 device, and the supernatant was discarded once clear. Washing was continued by adding 200 μl 1 X Stringent Wash Buffer (pre-heated to 47°C for 1 h) (Tube 4, SeqCap EZ Hybridization and Wash Kit) The sample was mixed by pipetting avoiding a major temperature drop and incubated for 5 min at 47°C. The tube was placed in the DynaMag-2 magnet, the liquid was discarded and the washing at 47°C with 1 X Stringent Wash Buffer was repeated once.

22: 200 μl 1 X Wash Buffer 1 (pre-heated to room temperature) was added to the Dynabeads plus bound DNA. The sample was vortexed for 2 min and the liquid was collected to the tube's bottom. Following magnetic concentration the liquid was discarded, and the sample was washed at room temperature with 200 μl 1 X Wash Buffer 2 (vortexing for 1 min) (Tube 2, SeqCap EZ Hybridization and Wash Kit), followed by a wash with 200 μl 1 X Wash Buffer 3 (vortexing for 30 sec) (Tube 3, SeqCap EZ Hybridization and Wash Kit) as described for washing with Wash Buffer 1. The tube was removed from the magnet, the bead-bound captured library was resuspended in 22 μl PCR-grade water and the entire sample (beads + liquid) was transferred to a 0.2 ml PCR tube.

**C PCR post-capture and sequencing**

23: An on- beads PCR amplification is undertaken to enrich library fragments, extend the adaptor sequence and incorporate an index to the P7 adaptor. The PCR reaction is using the KAPA® HiFi HotStart ReadyMix PCR Kit (KAPABiosystems, Boston, MA, Part # KR0370) in à final volume of 50 µl with:

15 pmol of SOL-PE-PCR_F primer (1)

aatgatacggcgaccaccgagatctacactctttccctacacgacgctcttc

15 pmol of SOL-MPE-INDX_R indexed primers CAAGCAGAAGACGGCATACGAGATXXXXXXGTGACTGGAGTTCAGACGTGT

This primer carries 6 bases of the official TruSeq Illumina Index.

Thermocycling parameters: 2 minutes at 98°C, followed by 18 cycles of 20 seconds at 98°C; 30 seconds at 62°C and 30 seconds at 72°C, with a final elongation of 5 minutes at 72°C. The reaction volume is 50 µL. A clean-up step is performed with 1.8x volume Agencourt AMPure XP magnetic beads. The elution volume is 20 µL.

24: Indexed libraries are individually controlled (sizing and estimation of the concentration) by electrophoresis on an AATI Fragment Analyzer™ device with the DNF-474 High Sensitivity Fragment Analysis Kit.

25: Three indexed libraries, corresponding to 144 captured barcoded DNA samples, are equally mixed. The final pooled library is quantified by qPCR with the KAPA Library Quantification Kit (Part # KK4824) and provided to the Get-PlaGe core facility (GenoToul platform, INRA Toulouse, France http://www.genotoul.fr) for sequencing.

26: The final pooled library is sequenced using the Illumina paired-end protocol on a single lane of a HiSeq3000 sequencer, for 2 x 150 cycles.

**SI References**

1. M. Mascher, et al. Barley whole exome capture: a tool for genomic research in the genus Hordeum and beyond. *The Plant Journal* **76**, 494-505 (2013).
2. N. Rohland, D. Reich D. Cost-effective, high-throughput DNA sequencing libraries for multiplexed target capture. *Genome Research* **22**, 939-946 (2012).
3. M. Mollion et al. Patterns of Genome-Wide Nucleotide Diversity in the Gynodioecious Plant *Thymus vulgaris* Are Compatible with Recent Sweeps of Cytoplasmic Genes. *Gen Biol Evol* **10**, 239–248 (2018) <https://doi.org/10.1093/gbe/evx272>
4. J. Fox, S. Weisberg. *An R Companion to Applied Regression,* 3rd Edition. (Sage Thousand Oaks CA, 2019)
5. G. James, et al. *An Introduction to Statistical Learning with Applications in R.* (Springer-Verlag New York, 2013).
6. Holtz Y, Ardisson M, Ranwez V, Besnard A, Leroy P, Poux G, et al. 2016 Genotyping by Sequencing Using Specific Allelic Capture to Build a High-Density Genetic Map of Durum Wheat PLoS ONE 11(5): e0154609 https://doi.org/10.1371/journal.pone.0154609
